# From Population Databases to Research and Informed Health Decisions and Policy

**DOI:** 10.3389/fpubh.2017.00230

**Published:** 2017-09-21

**Authors:** Yossy Machluf, Orna Tal, Amir Navon, Yoram Chaiter

**Affiliations:** ^1^Independent Researcher, Rehovot, Israël; ^2^The Israeli Center for Emerging Technologies (ICET) in Hospitals and Hospital-Based Health Technology Assessment (HB-HTA), Assaf Harofeh Medical Center, Zerifin, Israel; ^3^Sackler Faculty of Medicine, Tel Aviv University, Tel Aviv, Israel; ^4^Israeli Center for Technology Assessment in Health Care (ICTAHC), The Gertner Institute for Epidemiology and Health Policy, Tel Aviv, Israel; ^5^The School of Social Sciences and Humanities, Kinneret College, Sea of Galilee, Jordan Valley, Israel

**Keywords:** medical database, population-based research, evidence-based decision-making, comorbidity index, public health policy

## Abstract

**Background:**

In the era of big data, the medical community is inspired to maximize the utilization and processing of the rapidly expanding medical datasets for clinical-related and policy-driven research. This requires a medical database that can be aggregated, interpreted, and integrated at both the individual and population levels. Policymakers seek data as a lever for wise, evidence-based decision-making and information-driven policy. Yet, bridging the gap between data collection, research, and policymaking, is a major challenge.

**The model:**

To bridge this gap, we propose a four-step model: (A) creating a conjoined task force of all relevant parties to declare a national program to promote collaborations; (B) promoting a national digital records project, or at least a network of synchronized and integrated databases, in an accessible transparent manner; (C) creating an interoperative national research environment to enable the analysis of the organized and integrated data and to generate evidence; and (D) utilizing the evidence to improve decision-making, to support a wisely chosen national policy. For the latter purpose, we also developed a novel multidimensional set of criteria to illuminate insights and estimate the risk for future morbidity based on current medical conditions.

**Conclusion:**

Used by policymakers, providers of health plans, caregivers, and health organizations, we presume this model will assist transforming evidence generation to support the design of health policy and programs, as well as improved decision-making about health and health care, at all levels: individual, communal, organizational, and national.

## Introduction

In the era of big data, the medical community is inspired and required to maximize the benefit of processing the rapidly expanding medical datasets for basic, clinical-related, and policy-driven research. A comprehensive electronic personal medical record of individual patients should be available for physicians as a platform for complicated case management (1). Such patient-oriented activity can be bundled together with population-based activities, where individuals’ pieces of medical data or unprocessed facts can be aggregated (raw *data*), interpreted and integrated (*information* is meaningful rationally connected data), understood, enriched, and joined by semantics, experiences, and context (useful *knowledge* comes from finding patterns within information), delivered as actionable information (*wisdom* is understanding and application of knowledge), and drive *insights* (awareness of the underlying essence of the truth) at both the individual and population levels. Policymakers seek data as a lever for wise, evidence-based decision-making and information-driven policy.

However, the medical research enterprise and national health-care systems often operate as separate and almost non-related entities. Moreover, each acts as a closed club, therefore, sharing information, generating common insights, constructing and implementing high-quality evidence-based intervention programs and health-care plans are rare. The main challenges are (A) integration of (high quality and validated) data for the benefit of all relevant partners and (B) transforming these huge processed data into accessible and meaningful information for health-care leaders, to be wisely used for guidelines and policy, and for clinicians, to drive health-care decision-making. These involve legal, regulatory, ethical, financial, technological, and other aspects.

In the recent decade, the need for closing the evidence gap and building a strong foundation for the implementation of an evidence generation system to support a learning health system was recognized, and key principles and foundational elements were proposed (2). This need is separated yet complementing to the need of translating research findings into practical, useful, implementable interventions—the so-called “implementation science” (3). By creating an evolving scheme, we propose a model that bridges the gap between data collection, research and policymaking, spotlighting somewhat less attractive, though valuable, sources and routes of generating medical evidence, medical databases, and epidemiological studies, respectively. We developed a novel, modified, and multidimensional set of criteria to estimate the risk for future morbidity and mortality based on current medical conditions. It can transform evidence generation to support the design of health policy and programs as well as improved decision-making about health and health care, at all levels: individual, communal, organizational, and national.

Key steps of the model will be described, and examples from the Israeli health-care domains will illuminate the feasibility, applicability, and possible contribution of the model. The Israeli public system of health care—its characteristics, advancements, challenges, and opportunities—were described in details elsewhere (4, 5). With its many start-up life science enterprises (6) and digital health companies (7), Israel can be considered as a hub for digital health innovation. Balicer and Afek (7) recognized Israel’s four Is as core attributes: (I) information technology infrastructure and historical data repositories; (II) integration of care and data with interoperable data flow; (III) innovative spirit and supporting ecosystem; and (IV) implementation-ready providers and the way incentives are well aligned. These attributes, among others, are also central to our model, which provides a boarder, systemic, and applied perspective.

The relevance of the model is neither restricted to Israel nor to boundaries of a specific country. By the end of this article, the reader will be able to appreciate the significance of collecting, sharing, and analyzing (big) data (by policymakers, providers of health plans, caregivers, and health organizations) not only for better understanding the health and disease status of subpopulations and revealing risk factors, hidden associations and macro trends, but also for translating such insights into actionable health policy that may affect individual patients.

## The Model

The proposed model includes four steps of applicable implementation:

The first step is to gather all the relevant parties together in a task force and to declare a national program to promote collaborations, to support data and resources sharing, generation and evidence, and health policy and decision-making.
The second step is to promote a national records project that synchronizes the different records on both data and research levels, as well as increases accessibility and transparency. Cooperation on these two levels could create a two-way model, which would consequently create a continuum of information from infancy to adulthood and old age.
The third step is to expand the analysis of the organized and integrated data to pave the way for professional leaders to initiate fruitful interventions, by highlighting premorbidity findings in a disease-free population. Channeled research may facilitate this phase.
The fourth step, evidence-based decision-making, could encourage policymakers, operating in an environment of scarce resources, to estimate benefits and costs for comparative interventions and establish a wisely chosen national policy.

**Figure 1:**
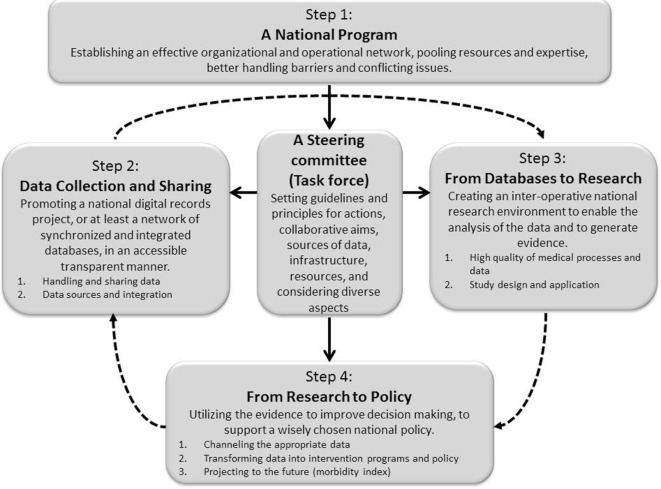
Schematic representation of a four-step model, aimed at bridging the gaps between data collection and sharing, research, data analysis, and policy making. Solid lines indicate direct relationship (such as initiating, directing, connecting, synchronizing, coordinating, monitoring, managing, etc.) between process components or steps of the model, while dashed lines indicate a non-direct relationship (such that utilizing “outcomes” of the former prerequisite step).

Schematic representation of the model is provided in Figure 1.

See Box 1 and Table 1 for key elements for implementation of this model and realizing the full potential of population databases for research and informed health decisions and policy. As will be illustrated below, this model also addresses the key perceived and emerging barriers of health policy makers concerning their use of evidence: decision makers’ perceptions of research evidence, absence of personal contact and gulf between researchers and decision makers, lack of timeliness or relevance of research, mutual mistrust, practical constrains, and power and budget struggles—therefore, it may improve appropriate use of research evidence in policy decisions (8, 9).

Box 1 **Key elements for realizing the full potential of population databases for research and informed health decisions and policy**

In the modern era, it has become essential to generate high-quality evidence to support improved health-care decisions and design of health policy. Nevertheless, while diverse resources of digital data are available, creating the environment that would enable research to generate evidence and transforming such evidence into decision-making and policy is still a challenge. To this end, numerous key elements are proposed (see details in Table 1):

- Establishing a national program, led by a multidisciplinary professional steering committee, to pave the way toward this goal.
- Setting guidelines and principles for data collection, sharing, and integration, while referring to collaborative aims, sources of data, infrastructure, resources, and considering legal, regulatory, ethical, financial, technical, technological, and other aspects.
- Establishing an interoperative national research environment to enable the analysis of the organized and integrated data and to generate evidence.
- Estimating the risk for future morbidity and mortality, using the suggested matrix, in order to prepare an evidence-based future health plan while considering both clinical and economical manifestations.
- Drawing guidelines to support health decision-making, and designing (and implementing) preventive and intervention programs—to improve health care.

Such a comprehensive and integrative action might enable the recruitment and commitment of the relevant parties, the establishment of a research environment, the generation of high-quality evidence, and its transformation to supporting informed health decisions and policy.

**Table 1:** Key elements for information-driven policy—recommended components, objectives, stakeholders and partners, methods, and targets.

Step in model	Element (component)	Objective (aim)	Who (stakeholders and partners)	How (methods)	Why (target)
Step 1	Establishing a national program	Initiating, directing, coordinating, monitoring, and managing the process	Governmental health and regulatory agencies, policy makers, medical institutions, physicians, researchers, epidemiologists, data analysts, information technologists, insurers, suppliers, business and third sector organizations/foundations, private sector, and public officials	Discussions within a multidisciplinary steering committee, producing guidelines and positional papers	Setting short- and long-term goals, building an integrated plan (taking into account technical, clinical, legal, ethical, methodological, etc., aspects) and operational network, recruiting and allocating resources and expertise, removing barriers

Step 2	Setting principles for data collection, sharing, and integration	Connecting, synchronizing, synthesizing, integrating, and sharing biomedical data	Insurers, suppliers, equivalent of IDF’s medical database, social security, research entities, MOH, governmental authorities, national registries, information technologists	Either a national record project or a network of databases or “research rooms”—providing unidentified/anonymized information for research and policy as well as identified information for intervention programs	Creating routes to share data and integrate complementing databases as a basis for research to generate evidence to support policy design and decision-making, as well as to improve interventions at all levels: individual, regional, and national

Step 3	Establishing a research network to realize the full potential of the databases	Conducting research to assure high-quality medical processes and to generate high-quality evidence, *via* diverse study designs that are the basis for drawing recommendations and guidelines (as part of policy or driving decision-making)	Policy makers, researchers, epidemiologists, physicians, public	Removing technical, organizational, and cultural challenges to allow collaborations and conducting diverse study designs, including observational studies (and randomized control trials etc.) to reveal the prevalence/incidence of medical conditions, secular trends, associations with socio-demographic variables and other medical conditions (“medical signatures”)	Estimating the clinical burden and subsequent functional disability → prioritizing mode of action and preferred medical topics

Step 4	Estimating the risk for future morbidity	Projecting from current health status (odds/risk/hazard ratio for morbidity or mortality) to future status and required health service demands	Policy makers, health service providers, researchers, epidemiologists, suppliers, insurers	Adaptation and utilization of the morbidity index/matrix, while using the “medical signatures” of specific subpopulations at risk (outcomes of step 3’s studies)	Preparing an evidence-based future plan while considering both clinical and economical manifestations, designing and implementing preventive and intervention programs
	Designing and implementing intervention programs	Transforming data into intervention programs and policy aiming to improve health condition and health care at all levels	The steering committee, epidemiologists, physicians, policy makers	Educational/preventive/monitoring (screening)/and intervention (treatment, preferred personalized medicine) activities among subpopulations at risk	To reduce morbidity and mortality, to improve health status and quality of care, to support decision-making and design of health policy

Hereafter, the rationale for each step of the model will be described, as well as its main issue/s, or fundamental aspect/s. For each issue, the main challenges will be portrayed, as well as the current status and actions taken toward the desired status (current opportunities), and key elements for successful implementation and its application (recommended future directions).

### Step 1: Establishing a National Program

#### The Rationale

Promoting the development, integration, and usage of (big) health data, in local or national health-care systems, cannot take place without a visionary health-care leadership endorsing the proper policy, or at least promoting the necessary steps (top–down approach). Public health officials have great interest in promoting health and research, and medical and health databases should be seen as national resources. It is a responsibility and duty to optimally exploit these databases for the benefit of all relevant parties.

At the decision-making level—to design health policy, to monitor its effects, and to identify policy problems and their complexity—the state is expected to combine forces and merge databases, which will necessarily improve citizens’ standard and quality of life (10) and result in higher accountably for healthy behavior.

#### Challenges

Cooperation between the relevant public agencies, including governmental health and regulatory bodies, scientific enterprises, funding agencies, and other stakeholders, is still a challenge in Western countries (11–13) and also in Israel. Bringing all parties together is just a prerequisite, as they mainly need to act together to achieve a common goal, in an environment and culture that require combining expertise and resources.

#### Current Opportunities and Recommended Future Directions

This should be done under the umbrella of a national program that is managed and operates *via* a steering committee including members of all relevant parties (from governmental agencies, to academic and medical institutions, the private sector, etc.). The distinctive national initiative “Digital Israel” can serve as a model. This project is aimed to set in motion a coordinated, effective effort to realize the potential of advanced technological infrastructures and informatics, and particularly to pave the way for a strategy for regulating and sharing health data as well as designing a national and uniform electronic medical record. The project—through a taskforce including governmental departments, local authorities, academic and health agencies, businesses, and third sector organizations–strives to enable the analysis of health information produced in the health system for research purposes, using advanced tools and big data technology, to build a national platform for research-intensive health-care information system. The National Council on Digital Health and Innovation is a multidisciplinary advisory entity to the Ministry of Health in areas related to opportunities arising from the digital world, technology and innovation in health, and harmonizing their coordination and implementation at all levels (14).

A national program, with short- and long-term goals, may establish an effective organizational and operational network, pooling resources and expertise, better handling barriers, and conflicting issues. Above all—each party can see its own and others’ contribution and benefits as part of a national act.

### Step 2: Data Collection and Sharing

#### The Rationale

European information sharing initiatives have already been proven beneficial in terms of patient safety and care (15, 16). Integrating medical knowledge and clinical data in a cooperative manner may support health-care decision-making (17). Diverse projects in the US “are already using digital data from the clinical settings to generate meaningful evidence that is needed to support informed decisions about health and health care” (2). Driven by ethical and medical justifications, professional incentive to improve quality of care and outcomes at both the personal and community levels, as well as the wish to contribute to patient satisfaction, a call to collect, connect, synchronize, synthesize, integrate, and share biomedical and physiological data is only natural.

Three key issues of “data collection and sharing” are reviewed: (1) handling and sharing data; (2) data sources and integration; and (3) adolescents’ health status as a baseline for a national health database.

#### Issue#1: Handling and Sharing Data

##### Challenges

It is noteworthy that concerns over privacy, confidentiality and data security, control and ownership of data about individuals, transparency of interests and processes, regulations, responsibility, and accountability should all be considered with the proper degree of caution and rigor (18–20).

##### Current Opportunities

Many of the legal, ethical, technological, managerial, and regulatory aspects were recently discussed at national (21) and international (22) conferences of the Israel National Institute for Health Policy Research (NIHPR).

The Patient’s Rights Act provides and allows a legal possibility to transfer medical information between agencies, and the Freedom of Information Act establishes the right of every citizen or resident to receive information held by public authorities, including for research purposes. Most researchers are not aware of this legal right. In addition, most patients are not just unable to express their desire or wish for use of their medical data, but are not even aware of the barriers for utilizing their medical data, and its potential beyond the direct clinical need of treatment.

##### Recommended Future Directions

It is essential to establish mechanisms to promote public awareness, understanding, and cooperation as well as patient consent, especially if information sharing is to be in the opt-out mode. In parallel to the abovementioned regulatory legal-legislative issues, a complementary ethical code should be formulated. It should deal with privacy and data security, benefit and harm, reciprocity, equality, transparency, rights of first use of the information, and fairness.

In addition, in light of ethical, commercial-economic, legal and professional projections for transferring and sharing medical information, the possibility to establish a mutual central database (either including individuals’ identifying details or an anonymous one) is less favorable and was rejected by working expert teams at these conferences. Rather, creating a legal, scientific, and cultural infrastructure for collaboration within the public system (decentralized network model), under governmental lead and regulation, was proposed. As an alternative, the establishment of “research rooms” in all or every organization has been proposed. These will enable external researchers to access information and analyze it on the enterprise platforms, extracting only the aggregate/statistical/de-identified outputs rather than raw data.

#### Issue#2: Data Sources and Integration

##### Challenges

An enormous amount of medical data is accumulated throughout peoples’ life; various medical reports, imaging and laboratory data are gathered in the health system, complementing surveys and registries on medical and lifestyle behavior, etc. Yet, all these data sources are not synchronized, and serve different agencies that act separately.

##### Current Opportunities

Presently, in Israel, numerous big databases—from military recruiting offices to public health services [general hospitals, Health Maintenance Organizations (HMOs), insurers, and private medicine facilities]—hold a great deal of data regarding individuals’ health and disease status. The Israeli medical system is still attempting to harmonize and integrate the various and heterogeneous databases. An integrated hospital-community online medical information system (OFEK virtual medical records) was established a decade ago, enabling physicians to observe medical findings and improve quality of care and medical service (23–25). Another example of integrating data from different sources is the National Program for Quality (Medical) Indicators in Community Healthcare, initially defined as a research project and then extended to a national project (4). HMOs, public hospitals, the Ministry of Health, and the NIHPR join together to regularly examine community health system performance in a variety of areas based on a set of indicators that are continuously updated. Furthermore, the Ministry of Health currently consolidates national registries (cancer, dialysis, diabetes, etc.) that involve transferring information from HMOs, hospitals, and private medical institutions. In addition, a nationwide pilot project for information sharing and cooperation between all players in the health-care arena: primary care clinics, the Ministry of Health, the National Insurance Institute, the Israeli National Cancer Registry, and the Ministry of Social Affairs and Social Services, has been running for several years (26).

##### Recommended Future Directions

These programs and their outcomes indicate the feasibility and contribution of cooperation between diverse agencies and data sharing. Thus far, it is mainly used to analyze performance at the system or national level. It should be extended in terms of the number of agencies cooperating, the type of data shared, and the purposes the shared data serve, mainly to inform decisions also at the individual level.

#### Issue#3: Adolescents’ Health Status As a Baseline for a National Health Database

##### Challenges

In Israel, where military service is compulsory by law, a distinctive opportunity to collect baseline medical data from medical examinations at recruitment centers enables us to learn about the health status of the bulk of the general young population (over 70% of the population), adolescents at the age of 16–19 in a premilitary service status (27). The thorough and comprehensive medical process at the army recruitment centers generate a massive database, which is only seldom viewed as integral to “non-military” health medical databases, as information sharing is only anecdotal rather than systematic.

##### Current Opportunities

The medical process at the recruitment center involves preliminary documentation from the primary care HMO physician, examination of anthropometric measures, urinalysis and visual acuity testing, a systematic and thorough anamnesis including family history, habits, and psychological evaluation, and a complete physical examination and referral if necessary for further investigation according to findings. Finally, a 5-digit profile and appropriate Functional Classification Codes [FCCs, which are similar to the International Classification of Disease (ICD) coding] that describe medical status (anatomical site, condition and severity, as well as an element of occupation) are assigned to each recruit and stored in a computerized database. This provides a transparent standardized record of “health status” of each individual for other physicians, whereby the rank of disability enables limiting activity by the non-medical commanding sector while the medical condition remains obscure (26–30).

##### Recommended Future Directions

Synchronizing all medical records—to allow continuity of medical data and information along the lifespan of individuals, and providing access to the accumulating data to the relevant parties in a multilateral manner—is pivotal to closing the evidence gap. The thorough and comprehensive medical process at the army recruitment centers, generates a massive database that provides an opportunity to assess heath status among adolescents, and may serve as a platform for planning preventive, educational, and medical intervention programs to improve current health-care services and reduce future illness (26).

### Step 3: From Databases to Research

#### The Rationale

Beyond the importance of digital data, including the army medical database, for individual treatment continuity, investigating the accumulative data of many individuals can serve to both test health-care policy-derived questions and to generate evidence (2) that in turn may affect policy, in a bi-directional manner. Such data investigation can serve for management, control, and quality assurance of medical processes and informatics (30, 31), but also for identification of morbidity patterns, mapping of health behavior, and characterization of epidemiological trends (25, 26). This and other issues are related to the role research plays (in the operational, implementation, and health system domains) in strengthening health systems, improving system performance and public health impact (32), as well as the complex interactions between health and health care (33). Establishment of a virtual research network is central to the activity of the national program and may rely on experience and benefits of already established ones, such as the HMORN (34–37). Creation of an interoperative national research environment entails substantial technical, organizational, and cultural challenges (2). Hereafter, the army medical database and its utilization in research will serve as a model.

“From databases to research” is examined through two main issues: (1) high quality of medical processes and data and (2) study design and application.

#### Issue#1: High Quality of Medical Processes and Data

##### Challenges

Emphasis on standardization of data collection and diagnostic criteria, as well as quality control and assurance of the medical processes that generate the (presumably valid, reliable, and high quality) data (30) are prerequisites for any research that is aimed at generating evidence to support health-care decisions and the design of health policy. Assuring such high-quality data is not trivial and poses a main challenge.

##### Current Opportunities and Recommended Future Directions

The medical database (alongside other resources) is central to the activity of a quality assurance and control team (38), which operates *via* an analysis → design → implementation → evaluation → modification loop (26, 28, 29, 31). Based on insights and recommendations of this activity, targeted intervention programs were designed, implemented, assessed, and modified. These programs encompassed key aspects of training, supporting, and mentoring the administrative, technical, and medical staff to consolidate and enforce uniform standards and guidelines. Significant and long-lasting improvements were observed in the quality of the medical process (anamnesis and physical examination, diagnosis, discretion and decision-making, and documentation), data (in term of validity and reliability) and care, as well as in advancing the awareness, knowledge, skills, perception, and motivation of the staff (26, 28, 29, 31, 38).

#### Issue#2: Study Design and Application

##### Challenges

Approaches that leverage and extend the use of the volumes of relevant digital health and health-care data to facilitate efficient, streamlined randomized controlled trials (RCT), high-quality observational studies, and cluster-randomization designs, which in turn will be able to generate evidence that ultimately leads to improved health outcomes and a more efficient health-care system, were already proposed (2). While RCTs, though valuable, require diverse resources, alternative and complementary research designs may provide useful information (of different quality), with minimal resources.

##### Current Opportunities and Recommended Future Directions

Analysis of the database by FCCs and risk determinants was conducted by a multidisciplinary research team from the fields of clinical medicine, epidemiology, biostatistics, public health, quality control, and policy research. The most common and natural observational design for such a study would be retrospective cross-sectional surveys, though cohorts and case-control studies are also valid.

Such studies can deal with prevalence and incidence of the medical condition among selected populations, secular trends of certain medical conditions, possible associations with sociodemographic variables, and interrelations with different anthropometric indices and other medical conditions. Previous studies that explored the IDF medical databases, or selected subpopulations based on year of examination, gender, region, etc., have focused on medical conditions such as extreme body-mass index (27, 39–45), hypertension (46, 47), asthma and other common respiratory diseases (48–51), myopia (52), cardiovascular diseases (53), diverse orthopedic disorders (54–57), inflammatory bowel disease (58), and others (59). These medical data can be linked to other sources of information, for example: the Israel National Cancer Registry (60–69), the Israeli Treated End-Stage Renal Disease (ESRD) registry (70), and the Israel Central Bureau of Statistics (71).

Such information may enable the identification of specific subpopulations at risk for future morbidity and even mortality, given the background characteristics and “medical signature” [similar to the concept “genetic signature,” the term is used here to describe the pattern (or profile) of specific and detectable medical conditions and diseases]. It can also serve to design health-care plans (and even policy) regarding the subpopulation under investigation, as well as to design targeted preventive/treatment programs.

### Step 4: From Research to Policy

#### The Rationale

The importance of health research utilization in policymaking, and its possible contribution to policies and desired outcomes, is increasingly recognized (72). In the shift from an individual-clinical to a population-policy level, the decision-making process becomes more uncertain, variable, and complex. In evidence-based decision-making, the context impacts on what constitutes evidence and how that evidence is utilized (73). Individualized medical data combined with cost and outcomes data can “make health economic analyses more disease specific and population specific but may require new skill sets,” hence informing and impacting both health economics and public health policy (74).

“From research to policy” is reviewed through three key issues: (1) channeling the appropriate data; (2) transforming data into intervention programs and policy; and (3) morbidity indices.

#### Issue#1: Channeling the Appropriate Data

##### Challenges

These days the challenge is channeling data and assembling information toward applied knowledge-based policy. Researchers provide and share outcomes of their studies to create a rational platform to support a preferred intervention alternative, yet, policymakers cannot always access and utilize these conclusions as evidence to approve policy. On the other hand, the wealth of data tolerates variant interpretations that may lead to scattered unfocused interventions.

##### Current Opportunities and Recommended Future Directions

To bridge these disparities, the methodology of “regulatory science” is applied, meaning focusing on policy-targeted literature evidence, with the purpose of thoroughly evaluating the most relevant data prior to decision-making (75). The development of an interoperable data network and an interoperative national research environment as part of a learning health system may also support this process (2).

It requires the collaboration and joined forces and resources of partners within the committee of the national program (Step 1), also including stakeholders and policymakers, physicians, researchers, data analysts, information technologists, insurers and suppliers, as well as representatives of the patients or the general public.

#### Issue#2: Transforming Data into Intervention Programs and Policy

##### Challenges

Gathering data, either from routine medical examinations or from research databases, may serve as a policy-driven force (76) to change health policy and clinical guidelines. This main challenge is how data regarding monitoring of specific conditions, and among specific subpopulations at risk, can be translated into conducting preventive, educative, and intervention programs reducing present and future morbidity and mortality, targeted screening for early detection and treatment, and planning of required resources and professional personnel for the health-care system.

##### Current Opportunities and Recommended Future Directions

Examples of dealing with the challenges are provided: (1) it was proposed that differences between Israeli Jews and Arabs in cardiovascular morbidity and mortality rates were potentially associated with dietary risk factors (77), which contributed to focused education and medical interventions in focus populations; (2) upward trends in the prevalence of both asthma symptoms and allergic diseases in Israeli adolescents (78) have resulted in a change in the screening exams carried out by family physicians; (3) reexamining the trends in breast cancer incidence, mortality, and survival in Israeli Jewish and Arab women (79) dictated a change in the actionable/operational guidelines (“individual marketing” within the Arab population on top of the guidelines at the community level) to fully realize the national policy of conducting mammography as a screening test; and (4) screening for and management of obesity and overweight in adults led to a diet intervention in a targeted population (80). The benefits and harms of screening and earlier treatment of overweight in children and adolescents in clinical settings have been deliberated in a recent evidence synthesis (81).

The significantly higher risks of current and future illness and death among overweight and obese adolescents (27) may lay the grounds for a preventive and interventional nationwide program. We suggest that such a program should include diverse complementing components, at the individual and community levels:

- General educational activities for promoting a healthy lifestyle to school students, their parents, and school personnel.
- Conducting repetitive surveys to screen for habits (smoking, alcohol use, drug abuse, etc.), physical activity, dietary and lifestyle scores, medical history, BMI, and risk indices such as blood pressure. This component may extend the current Israeli National Health and Nutrition Survey (82–84).
- Based on sociodemographic features, familial history of weight, and medical signature (27), one may construct profiles of populations at risk. This in turn may be utilized in assigning individuals to relevant specific programs, to identify, monitor, follow and treat weight problems and implications, in a personalized manner, also changing patients’ eating patterns (85).
- Specific subpopulations may require special tests to obtain comprehensive supporting data related to specific medical risk or tailored medical treatment. These may include, for example, candidate polymorphic sites or whole exome genotyping.
- Conjoining the national taskforce—to study, design, implement, and evaluate the policy and related program (in practice)—with the International Obesity Task Force, World Health Organization, Centers for Disease Control and Prevention, etc., to determine standard criteria (86–89), and adoption of global guidelines, such as in the case of gestational diabetes mellitus (90, 91).

Such a program may enable proper planning and provision of health-care services in the near future for the relevant subpopulation, and furthermore may contribute to reduce future associated morbidity and/or mortality. An “adapted” program can be designed for other medical conditions and targeted subpopulations, such as subjects with microscopic hematuria that are at high risk for ESRD (70).

Integrating demographic data with medical information may enhance efficiency in resource allocation, and translate into proactive interventions, especially in lower socioeconomic areas, which suffer decreased accessibility to health services. Insured adults in such areas are less likely to have blood tests, although suffering a higher prevalence of prediabetes and less frequent dietitian visits (among the tested patients) (92). Similarly, socio-demographic factors and primary health-care service utilization were found to be associated with reduced hypertension awareness and control. Specially focused outreach may be needed to improve hypertension awareness among minorities, certain subgroups not traditionally considered to be at high risk, and those who have less contact with the health-care system (93).

The other side of the coin is research that evaluates intervention programs and may affect policy design. For example, a unique randomized control trial revealed that a 12-month culturally sensitive intensive lifestyle intervention, compared to a moderate one, was effective in improving some of the metabolic syndrome components in obese non-diabetic Arab adult women (94). One may view health policy trials (95) as extension of such research.

#### Issue#3: Morbidity Indices—Looking Forward into the Future

##### Challenges

There are a few existing morbidity indices, most of which deal with coexistent illnesses and their impact on life span and mortality. Such is the Charlson Comorbidity Index, originally developed to predict survival (96), then adapted for predicting costs (97), and then used prospectively to identify patients who are likely to incur high costs in the subsequent year (98). Other adapted comorbidity indices are designed for and adapted to the ICD diagnosis and procedure codes, and are useful in studies of disease outcome and resource use employing administrative databases (99). Nevertheless, none of the existing morbidity indices addresses the impact of medical conditions encountered during adolescence on adulthood morbidity and mortality.

##### Current Opportunities and Recommended Future Directions

A score matrix that addresses the risk of future morbidity and mortality based on current medical conditions at adolescence can be a platform for treatment planning, further prioritization, resource allocation and policy outlines, as well as for research design. A modified set of criteria, or parameters, to estimate the risk for future morbidity is proposed as a multidimensional scale regarding a given medical condition, where a plethora of medical conditions can be related to the general healthy young population or specific subpopulations (defined by age, gender, origin, residential area, etc.):

- The current and future *prevalence* of the medical condition among the population. The higher the prevalence, the more subjects are affected and expected to be affected by the condition, respectively.
- The *odds/risk/hazard ratio* for morbidity or mortality. The higher the ratio, the higher the risk of developing the medical condition or dying from its implications.
- The *clinical burden* created by the grade of disease severity, which is best represented by *functional disability*, rather than a laboratory or imaging presentation. This would estimate the impact on life quality and expectancy, where death is obviously the ultimate worst outcome.
- The *time frame* for the occurrence of the medical condition; is it immediate (near future) or far (in the long term). The longer the time for the condition to develop, the longer the time for surveillance and taking preventive actions. Here, the time from diagnosis to advanced illness should also be taken into consideration.

**Figure 2:**
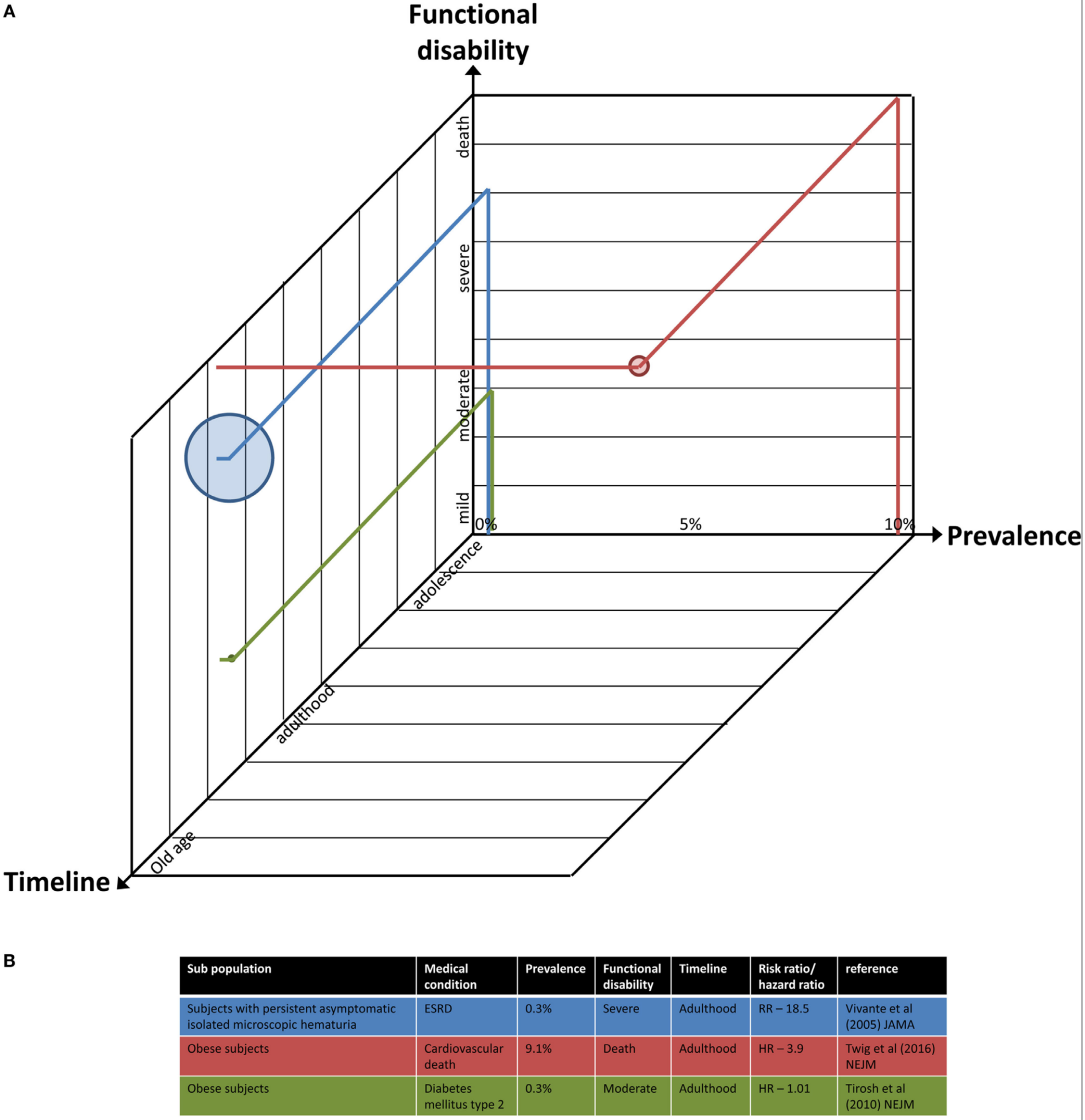
A multidimensional illustration of the parameters to estimate the risk for future morbidity. **(A)** The axes represent current/future prevalence of a medical condition among the population (percentage), time frame of its occurrence (adolescence to old age scale), and the estimated functional disability imposed by the grade of its severity (mild to severe and even death). The size of each point is proportional to the odds/risk/hazard ratio for morbidity or mortality. Projection of data of three exemplary studies into the illustration is provided. **(B)** Details of the data and studies used in panel **(A)**.

For example, the current prevalence (27, 39, 42) and future risk (46, 100) of diabetes and coronary heart disease among obese adolescents were calculated, as well as related adulthood mortality (101) and mainly cardiovascular death (71). In contrast, the presence of persistent asymptomatic isolated microscopic hematuria in 16- to 25-year-old subjects (diagnosed in 0.3% of eligible individuals) was associated with a significantly increased risk (around 19) of treated ESRD for a period of 22 years (70), a condition that confers significant functional limitation and is life threatening. An illustration of this data is graphically presented in Figure 2.

Combining these parameters, which are driven by analysis of databases and observational studies, results in a numerical value that estimates the level of risk for a given medical condition, on a 1–4 scale, from low to very high, respectively. It encompasses the four dimensions. The use of such a matrix is in a way reminiscent of the production and use of a set of evidence-informed recommendations for improving the discharge and post-discharge care of infants following intervention for congenital heart disease (102), although it was conducted on patients and not on a healthy population.

## Discussion and Concluding Remarks

### A Call for Action

A four-step model has been proposed, aimed at bridging the gaps between data collection and sharing, research, data analysis, and policymaking. In Israel, as shown above, several steps have been launched and are still ongoing and may lay the ground toward the realization and fulfillment of this model. Nevertheless, there is room to significantly expand these approaches and implement them on a broader scale and in diverse areas of medical processes, public health, and health-care policies supported by evidence, both in Israel and worldwide.

This novel comorbidity matrix may reflect both clinical and economic manifestations of a certain medical condition within a subpopulation. This, in turn, may lead policymakers to design a specific program to monitor and address the risk among the targeted subpopulation, and the primary physician to fulfill it. Moreover, such an index may inform and support decisions regarding prioritizing and planning of expected needs such as allocating medical services, purchasing facilities, recruiting personnel, and costs. The use of such a matrix may facilitate stewardship of medical pathways, both in individual treatment charts and decision-making as well as in population-based policymaking.

### Challenges and Barriers

The feasibility and applicability of the model were illustrated mainly from the perspective of the Israeli health system. It encompasses diverse and complementary medical databases, including the comprehensive IDF health database. The model is of high relevance to other countries across the world where similar resources (even if not identical) and medical processes exist, such as countries where military service is compulsory (e.g., Norway, Greece, Switzerland, Finland, Denmark, Brazil, and until recently also Sweden), countries where regional-level or nationwide screening processes (such as at schools) has been already established or will be initiated soon, and generally countries that collect digital medical data and strive to use it to generate evidence to support health-care decisions and health policy. Under ideal conditions, adjacent and cooperating countries (in the European union, Great Britain, and so on) could collaborate to gather and share big medical databases for the purpose of research, and design multi-intervention programs to improve the quality of life and life expectancy of their citizens. In reality, the politics, power relations, and narrow interests between diverse medical parties within a given country may hinder data collection and sharing, research, and transformation into policy design. Thus, a structured program may facilitate a successful national plan scheme.

To meet the need of using electronic health record systems as a basis and platform for designing wise policy and to improve population health, the quality of health care and the performance of health systems—the OECD published a thorough report, which summarizes the progress in OECD countries and challenges regarding: health informatics infrastructure, protection of privacy, regulations, collection and use of personal health data, data linkage and access, and above all—similarities and differences among countries’ data systems, challenges, and opportunities at the national levels (103). Most of these aspects have been deliberated here with regard to the proposed model, bringing to light both shared guiding principles and unique opportunities.

A fundamental problem with most data suppliers is the lack of incentives to provide data for research in general and in the field of policy specifically. Barriers are cost and complicated application processes that are driven by rigid rules that obstruct access to more affordable data from the health and social care data centers, owned by the government and commercial traders (104).

The growing aggregation of data stored as “big data,” requires complex analysis methodology to challenge the formulation of a fundamental process at the national level. It is not only the “technical” need to prepare the next generation of clinicians who “understand and participate in the enterprise of extracting lessons learned from digitally captured patient care” (105). In England, clinicians and suspicious patients must be convinced that the needs and benefits of collecting and using “big data” to help improve patient care, and make the national health system more efficient, outweigh the risks (106). Moreover, the governmental care data program, which plans to bring together health and social care data from primary and secondary care and make them available to “approved groups of users” (including researchers) has been criticized on grounds of data privacy, which has not only led to an extended delay in its implementation (107, 108), but also to the expectation that a large number of people will opt out (109). One must keep in mind that scattered, disorganized data may bias and become a hindrance, so that intensive, yet prudent, efforts should be considered while implementing such a move.

Funding this complex journey is still an open debate. Worldwide, electronic health records and health data exchange technologies are being implemented widely, despite limited empirical data and mixed results of their impact on ambulatory quality (110), due to investment by the governments in financial incentives for the adoption and meaningful use of such technologies (111, 112). Yet, the following key steps—such as maintaining and improving the databases, conducting research, reporting on findings, drawing and implementing policy and so on—should also be financially supported. Asking the individual patient to share the financial burden, either as an optional or a mandatory act, is questionable, as the patient has relatively low interest and minor incentives. Applying to other consumers of data (such as the pharma industry, insurance agencies, and the private sector) may assist in raising a budget, but has its own pitfalls, such as access to confidential medical data, risk of inappropriate use of the data, conflicting financial interests, etc.

The rationale and promise of accessible, reliable, and comprehensive medical databases have been recognized by policymakers. Nevertheless, the concept of sharing databases within the health-care system sprouts from the hypothesis that the relevant stakeholders have the need and are willing to transfer information. Creating a national taskforce may facilitate this mission. Other players, such as private insurers and the industry, may also have an interest in sharing these databases, and their incentives should be carefully discussed. In the era of transparency, the role of the patient has not yet been fully taken into consideration, and a survey to reveal the standpoints of the general public may contribute to broader understanding of a preferred strategy and the formation of an effective policy.

## Author Contributions

OT, YM, and YC envisioned the model to bridge the gap between data collection and policymaking, as well as the modified and multidimensional set of criteria to estimate the risk for future morbidity. YM conceptually and graphically designed the schematic presentations within this manuscript. All authors conceived the conception and design of this article and were involved in drafting the article, iterative critical revisions for important intellectual content, and final approval of the article. Therefore, each of the authors takes public responsibility for the content and agrees to be accountable for all aspects of the work including ensuring that questions related to the accuracy or integrity of any part of the work are appropriately investigated and resolved.

## Disclaimer

All authors were (OT) or are (YC, AN, and YM) members of the quality assurance and control committee, IDF Medical Corps. Nevertheless, this manuscript is a result of long-term discussions between the authors, and it was conceived and written as a shared work neither related to that committee nor on behalf of the IDF, and surely not as representing any official (or non-official) position of the army.

## Conflict of Interest Statement

The authors declare that there are no commercial or financial or any other relationships that could be construed or perceived by the academic or medical communities as representing a potential conflict of interest. The opinions expressed in this manuscript represent the consensus of the authors and do not necessarily reflect the formal position of the affiliated institutions.

## Abbreviations

CCI, Charlson Comorbidity Index; CDC, Centers for Disease Control and Prevention; ESRD, End-Stage Renal Disease; FCC, Functional Classification Codes; HMO, Health Maintenance Organization; ICD, International Classification of Disease; IOTF, International Obesity Task Force; SES, Socio-Economic Status; WHO, World Health Organization.
